# Adequacy of Antenatal Care at Ministry of Health Facilities in Jeddah, Saudi Arabia: A Cross-Sectional Study

**DOI:** 10.7759/cureus.61113

**Published:** 2024-05-26

**Authors:** Ali M Alsolami, Abdulmajeed G Alamri, Ali H Khodari, Raghda K Tayeb

**Affiliations:** 1 Public Health Administration, Ministry of Health, Jeddah, SAU; 2 Joint Program of Preventive Medicine Post Graduate Studies, Ministry of Health, Madina, SAU; 3 Obstetrics and Gynaecology, King Abdulaziz Hospital and Oncology Center, Jeddah, SAU

**Keywords:** saudi arabia, jeddah, neonatal health, apncu, antenatal care

## Abstract

Background

Most pregnancy-related complications and undesirable outcomes are preventable by effective interventions at a reasonable cost. These interventions are mainly deployed during the antenatal phase and are included under the umbrella of antenatal care (ANC). To our knowledge, no studies have been conducted to assess the adequacy of ANC in Saudi Arabia. This study aimed to measure and quantify the adequacy of ANC provided by the Ministry of Health (MoH) facilities in Jeddah and to determine potential factors influencing ANC.

Methodology

In this cross-sectional study, we used the Adequacy of Perinatal Care Utilization index to measure the adequacy of ANC. Data were collected from September 2023 to March 2024 in two randomly selected MoH hospitals by interviewing mothers and collecting data from medical records.

Results

A total of 303 mothers participated in this study. Mothers’ mean age was 31 years, and 50% of them had received higher school education. Prevalence of adequate ANC was 64.7%. There was a significant association between the adequacy of ANC and mothers’ level of education (p < 0.001), time taken to reach the nearest primary care center (p < 0.001), number of total pregnancies (p < 0.034), and the total number prenatal visits (p < 0.001).

Conclusions

This is the first study to shed light on the prevalence of adequacy of ANC in Saudi Arabia and its associated factors. This study would pave the way to investigate the adequacy of ANC on a national level and will aid policymakers in developing and implementing effective ANC preventive measures, hence helping improve women’s health and their babies.

## Introduction

In 2015, more than 300,000 women died secondary to pregnancy-related complications, and more than two million babies were stillborn [1,2]. Most of these complications and undesirable outcomes are preventable by effective interventions at a reasonable cost [3,4]. These interventions are mainly deployed during the antenatal phase and are included under the antenatal care (ANC) umbrella. The World Health Organization (WHO) defines ANC as “the care provided by skilled health-care professionals to pregnant women and adolescent girls in order to ensure the best health conditions for both mother and baby during pregnancy” [5]. Components of ANC include risk identification, prevention and management of pregnancy-related or concurrent diseases, and health education and health promotion [5]. Effective ANC leads to a reduction in pregnancy and perinatal morbidity and mortality by direct means such as early detection and treatment of complications, and by indirect means such as stratifying women according to their risk for complications [6].

There is a clear discrepancy in the provision and utilization of ANC both between countries and within countries. A 2016 US review of the National Vital Statistics System database, which maintains records of all registered births in the United States, revealed that, overall, 75% of pregnant women received adequate ANC. The review also showed that underprivileged populations had much lower access and provision of ANC [7]. In contrast, a 2017 study from Ethiopia estimated that only 3% of women surveyed received adequate ANC. Similarly, the study reported a clear difference between privileged, high-income populations and those who are disadvantaged [8].

Several factors have been described in the literature that seem to influence the adequacy and utilization of ANC. Demographic factors such as age, income, and level of education strongly correlate with the adequacy of ANC. Accessibility and methods of payment for ANC services are also strongly associated with the level of adequacy of ANC [7,8].

To our knowledge, no studies have been conducted to assess the adequacy of ANC in Saudi Arabia. Thus, this study aimed to measure and quantify the adequacy of ANC provided by the Ministry of Health (MoH) facilities in Jeddah and to assess potential factors influencing ANC.

## Materials and methods

This analytic, cross-sectional study was conducted in Jeddah, Saudi Arabia. Inclusion criteria encompassed any puerperal woman admitted to the maternity ward who gave birth to a newborn whose gestational age was 20 weeks or more and received prenatal care in Jeddah. The data collection occurred from September 2023 to March 2024 in two randomly selected MoH hospitals out of four MoH hospitals that contain post-delivery maternity wards.

Informed consent was taken from participants to enter the study. The data collection was conducted by direct patient interviews and supplemented with data collected from medical records. The interviews were conducted by trained medical staff. Finally, the data were compiled in a standardized Excel sheet.

Measuring and quantifying the adequacy of ANC is essential in exploring the relationship between ANC and pregnancy outcomes and assessing the availability and accessibility of ANC services. Several indices have been developed to quantify the adequacy of ANC. The most widely used and supported by evidence is the Adequacy of Perinatal Care Utilization (APNCU) index [9].

The APNCU index attempts to characterize prenatal care utilization on two independent and distinctive dimensions, namely, the Adequacy of Initiation of Prenatal Care and the Adequacy of Received Services (once prenatal care has begun). This two-factor index does not assess the quality of the prenatal care that is delivered, but simply its utilization. It categorizes levels of prenatal care, based on the recommendations of the American College of Obstetricians and Gynecologists, into the following four categories: Adequate Plus, prenatal care begun by the fourth month and 110% or more of recommended visits received; Adequate, prenatal care begun by the fourth month and 80%-109% of recommended visits received; Intermediate, prenatal care begun by the fourth month and 50%-79% of recommended visits received; Inadequate, prenatal care begun after the fourth month or less than 50% of recommended visits received.

We estimated the sample size for this study using the OpenEpi software [10], assuming a 50% prevalence of inadequate ANC in the absence of previous studies from Saudi Arabia, with a 95% confidence interval and a 10% non-response rate. The sample size was calculated based on an estimated 10,000 deliveries per year at MoH facilities in Jeddah.

A multistage random sampling technique was used in this study. Four MoH hospitals have fully operational obstetric units that serve the Jeddah population. Two hospitals were selected using the cluster random selection technique.

The collected data were analyzed using SPSS Statistics, Version 26.0 (IBM Corp., Armonk, NY, USA). Descriptive statistics were used to analyze the data, including means, frequencies, standard deviations, and ranges. Chi-square tests were used to evaluate the associations between categorical variables and the adequacy of ANC, and independent-samples t-tests were used to compare the means of continuous variables among the adequacy of ANC groups. The level of significance was set at a p-value <0.05.

We obtained ethical approval (code A01455) from the Ministry of Health Institutional Review Board in Jeddah, and informed consent was taken from participants. Only anonymous data were collected to ensure confidentiality.

## Results

A total of 303 participants were recruited for the study. The mean age of mothers was 31 years with a standard deviation of 6.5, and around 50% of mothers had received higher school education. About 54% of newborns were male. More than 96% of mothers were non-smokers and one-third were employed. Over 40% of participants reported that the nearest primary care center would take them more than a 30-minute drive, and the average total prenatal visits were six. Around 12.5% of newborns were admitted to the neonatal intensive care unit (NICU), of whom 45% were admitted because of prematurity. Detailed demographic data are presented in Table 1.

**Table 1:** Demographic characteristics of study participants. NICU = neonatal intensive care unit

Characteristics		Count (%)
Mother’s age, mean (SD)		31 (±6.5)
Smoking status	Smoker	7 (2.3%)
	Non-smoker	292 (96.4%)
	Ex-smoker	4 (1.3%)
Level of education	Higher	150 (49.5%)
	Secondary	126 (41.6%)
	Intermediate	14 (41.6%)
	Elementary	9 (3%)
	Illiterate	4 (1.3%)
Total number of prenatal visits, mean (SD)		6 (±4)
Timing of visit initiation (Month), mean (SD)		2 (±2)
Gender of newborn	Male	162 (53.5%)
	Female	141 (46.5%)
Birth weight (g), mean (SD)		2,741 (±533)
Gestational age at birth (weeks), mean (SD)		37 (±3)
Total number of pregnancies, mean (SD)		3 (±2)
Admission to NICU	Yes	38 (12.5%)
	No	265 (87.5%)
Employment Status	Yes	102 (33.7%)
	No	201 (66.3%)
Time to the nearest primary care center (minutes)	<10	97 (32%)
	11–20	64 (21.1%)
	21–30	15 (5%)
	>30	127 (41.9%)

Regarding the APNCU index, our study showed that the overall prevalence of adequate ANC was 64.7%, as shown in Table 2. Particularly, more than 35 % received Inadequate care, whereas 26% received Adequate Plus care.

**Table 2:** APNCU index. APNCU = Adequacy of Perinatal Care Utilization

Antenatal care adequacy	Frequency	Percent
Adequate Plus	78	25.7
Adequate	71	23.4
Moderate Care	47	15.5
Inadequate Care	107	35.3
Total	303	100

For testing the association of levels of adequacy of ANC, we considered classifying patients who received Adequate Plus, Adequate, and Moderate care ANC as Adequate ANC whereas participants who received Inadequate ANC remained as Inadequate ANC.

Chi-square tests were used to evaluate the associations between categorical variables and adequacy of ANC. There was a statistically significant association between NICU admission and adequacy of ANC (x2 = 3.868, p = 0.049), as shown in Table 3. Moreover, level of education is significantly associated with receiving adequate ANC (χ^2^ = 30.784, p < 0.001). Moreover, the time taken to reach the nearest primary healthcare center was significantly associated with ANC adequacy (χ^2^ = 92.275, p < 0.001). However, smoking status, employment status, and the gender of the newborn did not show a significant association with the adequacy of ANC.

**Table 3:** Demographic characteristics stratified by APNCU index. ^*^: independent t-test; ^**^: chi-square test. ANC = antenatal care; NICU = neonatal intensive care unit; APNCU = Adequacy of Perinatal Care Utilization

Characteristics		Adequate ANC	Inadequate ANC	P-value
		Count (%)	Count (%)	
Mother’s age, mean		31	32	0.368^*^
Smoking status	Smoker	5 (2.6)	2 (1.9)	0.305^**^
	Non-smoker	187 (95.4)	105 (98.1)	
	Ex-smoker	4 (2)	0 (0)	
Level of education	Higher	75 (38)	75 (70.1)	<0.001^**^
	Secondary	97 (49.5)	29 (27.1)	
	Intermediate	11 (5.6)	3 (2.8)	
	Elementary	9 (4.6)	0 (0)	
	Illiterate	4 (2)	0 (0)	
Total number of prenatal visits, mean		8	3	<0.001^*^
Visit initiation month, mean		2	4	<0.001^*^
Gender of newborn	Male	105 (53.6)	57 (53.3)	0.960^**^
	Female	91 (46.4)	50 (46.7)	
Birth weight (g), mean		2,747	2,730	0.129^*^
Gestational age at birth (weeks), mean		37	37	0.715^*^
Total number of pregnancies, mean		2	4	0.034^*^
Admission to NICU	No	166 (84.7)	99 (92.5)	0.049^**^
	Yes	30 (15.3)	8 (7.5)	
Employment status	Yes	59 (30.1)	43 (40.2)	0.076^**^
	No	137 (69.9)	64 (59.8)	
Time to the nearest primary care center (minutes)	<10	84 (42.9)	13 (12.1)	<0.001^**^
	11–20	54 (27.6)	10 (9.3)	
	21–30	15 (7.7)	0 (0)	
	>30	43 (21.9)	84 (59.8)	

Independent-samples t-tests were used to compare the means of continuous variables among the adequacy of ANC groups. Mothers who had four pregnancies on average were significantly found to receive inadequate ANC compared to those who had two or fewer pregnancies (p < 0.034). Furthermore, mothers who started their prenatal care in their second month of pregnancy on average were significantly found to receive adequate ANC in comparison to those who started their ANC in their fourth month on average (p < 0.001). In addition, there was a significant difference in average total prenatal visits between mothers who received adequate ANC and those who did not receive (p < 0.001). On the contrary, the mother’s age, newborn’s birth weight, and gestational age showed no statistically significant differences in their means among ANC adequacy groups.

## Discussion

This is the first study to determine the adequacy of ANC in Saudi Arabia. We used the APNCU index to measure ANC adequacy. These findings may contribute to determining crucial factors associated with ANC adequacy and implementing effective prenatal preventive measures. About 65% of our sample were shown to receive adequate ANC, while 35% received inadequate care. This finding is relatively lower than that reported in the United States at 75% [7]. However, it was noticeably higher than that found in Rwanda and Ethiopia at 43% and 3%, respectively [8,11].

In addition, our study found a significant association between educational level and ANC adequacy which has been reported in the literature. This was seen in a study from Ethiopia which found that women’s educational level was significantly associated with the adequacy of ANC [8]. In contrast, a study conducted in Iran found an insignificant association between educational level and ANC adequacy [12]. This implies that receiving proper education could contribute to utilizing prenatal care services efficiently.

Additionally, a statistically significant association was found between the time taken to reach the nearest primary care center and the utilization of prenatal care services. Similarly, Kennedy et al. found a significant association between the number of prenatal care visits and distance traveled in rural areas of Kansas, United States [13]. This may play a critical role in the number of prenatal visits leading to adequate ANC.

Furthermore, we found that admission to the NICU was associated significantly with the adequacy of ANC. Nearly half of the newborns in our study were admitted because of prematurity. This finding is consistent with a study done in Iran by Tayebi et al., who investigated the adequacy of ANC and pregnancy outcomes [14]. They found a significant relationship between ANC adequacy and preterm labor. This finding would encourage public health authorities to focus their efforts on educating women of reproductive age regarding receiving adequate ANC which would have a positive impact on pregnancy outcomes.

Our study had few limitations. First, a relatively small sample size may limit the generalizability of the study. Also, selecting one city and not including all facilities in the study may be another limitation. This study could be affected by recall bias among women who received adequate ANC due to the nature of the study design. As this is a cross-sectional study, we could only investigate association rather than causality. Future prospective cohort design should be considered to determine the causality of some variables on the adequacy of ANC.

## Conclusions

This is the first study to shed light on the prevalence of adequacy of ANC in Saudi Arabia and its associated factors. Although the prevalence of adequate ANC in our study was mildly high, we think it could have been lowered by the small sample size and including only two hospitals, which may affect generalizability as well. This study could pave the way to investigate the adequacy of ANC on a national level and will aid policymakers in developing and implementing effective ANC preventive measures. Future research should be conducted to explore other potential variables longitudinally that may contribute to ANC’s adequacy on a larger scale to help improve women’s health and their babies.
